# Prescription and dispensing guidelines in Lebanon: initiative of the Order of Pharmacists of Lebanon

**DOI:** 10.1186/s40545-020-00273-9

**Published:** 2020-11-06

**Authors:** Aline Hajj, Hala Sacre, Souheil Hallit, Rony M. Zeenny, Georges Sili, Pascale Salameh

**Affiliations:** 1 Laboratoire de Pharmacologie, Pharmacie Clinique Et Contrôle de Qualité Des Médicaments, Saint-Joseph University, Beirut, 1107 2180 Lebanon; 2 Faculty of Pharmacy, Saint-Joseph University, Beirut, 1107 2180 Lebanon; 3 Drug Information Center, Order of Pharmacists in Lebanon, Beirut, Lebanon; 4 INSPECT-LB, Institut National de Santé Publique, Epidémiologie Clinique et Toxicologie-Liban, Beirut, Lebanon; 5 Faculty of Medicine and Medical Sciences, Holy Spirit University of Kaslik (USEK), Jounieh, Lebanon; 6 Clinical Pharmacy Department, American University Beirut Medical Center, Beirut, Lebanon; 7 Faculty of Pharmacy, Lebanese University, Beirut, Lebanon; 8 Faculty of Medicine, Lebanese University, Beirut, Lebanon

**Keywords:** e-Prescription, Lebanon, Physician prescribing pattern, Guideline

## Abstract

**Background:**

In Lebanon, difficulties in accessing medications are due to two main barriers, mainly: high cost and the lack of medication safety, related to poor-quality (irrational) prescription and use. The objective of this work is to suggest guidelines to implement a unified medical prescription in Lebanon. These guidelines are expected to promote medication safety and decrease pharmaceutical expenditures in the Lebanese context.

**Methods:**

The Order of Pharmacists of Lebanon (OPL) developed a comprehensive set of guidelines for physicians and pharmacists, including a detailed workflow process to improve the use of the unified medical prescription. The guidelines were presented to the Lebanese Ministry of Public Health (MOPH).

**Results:**

The project covered prescription guidelines to physicians (handwritten and electronic-prescriptions), and medication dispensing and generic substitution guidelines to pharmacists. Prescription guidelines included all required information about both the prescribing physician and the patient with the maximum of details, comprehensibility, and caution regarding specific populations/co-morbidities/co-prescriptions. Dispensing guidelines included details for safe and appropriate treatment dispensing, pearls for medications’ counseling and generic substitution, as well as specific consideration for at-risk populations or those with concomitant medications and co-morbidities. Finally, a suggested workflow clarified the process for improving the unified medical prescription.

**Conclusions:**

The implementation of the guidelines should now be formally evaluated, to assess if they achieve the aims to reduce prescribing and dispensing errors, to improve the quality of medicines' prescription and use, the patient care, and the interaction between all stakeholders

## Main text

### Background

Affordable and safe access to quality medicines is at the core of successful health systems; the World Health Organization (WHO) encourages countries to develop evidence-based policies and good governance throughout the supply chain, from selecting the right products to using them correctly [1–3]. In Lebanon, difficulties in accessing medications owe to two main barriers, mainly: high costs and the lack of medication safety [4–6].

The pharmaceutical sector in Lebanon is based on imported medicines predominantly (more than 80% of the total market share) [6], and that over 25% of annual household expenditures are spent on medications [6]. Consequently, Lebanon’s pharmaceutical spending per capita is among the highest in the Middle-East [7], and only around 20% of drugs used are reimbursed through public or private insurance [7].

Medication safety is another significant public health concern, particularly in a country where medication-related knowledge and patient interactions with primary caregivers are suboptimal [8]. Therefore, medication errors and adverse drug reactions can be distressing to both the patient and health care providers, causing severe medical and economic harm [9–11]. While adverse drug reactions are sometimes difficult to avoid, a medication error is usually avertible and defined as “any preventable event that may cause or lead to inappropriate medication use or patient harm while the medication is in the control of the health care professional, patient, or consumer” [12]. Some of these medication errors are seen at the early stages of medication use, particularly prescribing and dispensing, involving both the physician and the pharmacist [9].

Multicomponent interventions have been suggested worldwide to improve and secure access to medicine, improve the quality of prescribing/dispensing, and decrease pharmaceutical expenditures [9, 13]. As one of these strategies, the Lebanese Ministry of Public Health (MOPH) adopted in 2015 a unified medical prescription form among physicians, pharmacists, and patients (ministerial decision no. 1295) as a mean to promote generic drug use. The form, composed of several sections, targets physicians, pharmacists/radiologists/medical laboratories, insured patients, and third-party payers, but lacks essential fields such as age, gender, and diagnosis.

In this context, a study published in 2017 [14], exploring community pharmacists’ views and reported practices about the new generic drug substitution policy, revealed that pharmacists did not perceive a significant decrease in overall patient expenditures, nor an increase in generic drug dispensing. The authors also outlined a poor adherence to the policy from all stakeholders, including pharmacists, patients, and physicians; the latter seemed to overuse the option of non-substitutable medications [14]. Finally, the absence of an electronic prescription and patient profile platform magnify the risk of error. Hence, a complete medical record/tracking option would allow the pharmacist to give their best medication counseling [15].

To address all aforementioned issues, the Order of Pharmacists of Lebanon (OPL), the official professional association of pharmacists in Lebanon, suggested a comprehensive process to implement the unified medical prescription in Lebanon. The objective of this work is to present the suggested guidelines and processes, which implementation is expected to reduce the risk of errors at the physician prescribing and medication dispensing levels.

### Methods

#### Generating the guidelines

The scientific committee of the OPL worked to have clear guidelines for the application of the unified prescription policy, as previously decided by the MOPH. These guidelines are part of a governance framework of the health-system developed by the OPL, and inspired by the World Health Organization (WHO) [16]; their development took place through several steps.

#### Literature review

After a thorough literature desk-review, several internationally published documents were selected as adaptable to the Lebanese system. Out of dozens of documents found, the following were selected: Guide to Good Prescribing written by the WHO [17]; Guidelines for Dispensing of Medicines by the Pharmacy Board of Australia [18]; How to Write a Prescription: Guidelines for Dentist by the Irish Health Protection Surveillance [19]; General Prescribing Guidance by England National Health Service [20]; Writing a Prescription: Guidelines by Indian Medical Dialogues [21]; Appropriate Prescribing of Medications: An Eight-Step Approach by American researchers [22]; and Guideline for generic substitution-KNMP from Switzerland [23]. These documents were chosen since they were issued by international and national authorities and researchers such as American, Australian, European and India (a developing country).

#### Synthesis into four documents

The members of the scientific committee synthesized the literature and generated four documents. It is noteworthy that the committee is composed of pharmacists practicing in different settings: two community pharmacists, one clinical pharmacist, three academic pharmacists, and one managerial pharmacist.

After a series of meetings dedicated to this purpose, the committee submitted a draft to the OPL Board (elected members representing all pharmacy practice settings, i.e., community pharmacists, managerial pharmacists, marketing, and sales pharmacists) for review and approval. Two meetings were necessary to produce the final version of the three-part guidelines and the workflow process. They stated different points to the concerned parties:

1. To the physicians: Guidelines for writing prescriptions and E-prescriptions, and recommendations for the use of internationally approved abbreviations that are easy to read by the pharmacist (Additional file 1).
2. To the pharmacists: Guidelines for accurate prescription reading, safe medication dispensing, and generic substituting (Additional file 2).
3. To the MOPH: Suggested changes in the format of the unified medical prescription for better traceability, convenience, and ease of use (Additional file 3).
4. Workflow: Clarified the interaction between stakeholders throughout the process (Additional file 4).

#### Submission to concerned authorities

The OPL submitted a comprehensive project to the MOPH, including the guidelines, the changes suggested to the MOPH, the workflow process, and suggestions to improve the use of the unified prescription. The implementation of guidelines is expected to be soon, given that the MOPH administration welcomed the project and started to gather stakeholders’ opinion about it; however, various factors can influence and delay this process, including political turmoil, economic difficulties, the rigidity of the healthcare system, and the willingness of healthcare professionals to collaborate.

### Results

The details of the guidelines are presented and discussed below.

#### Handwritten and electronic e-prescription guidelines for physicians (details in Additional file 1) (24)

The guidelines include the basic legal requirements of a prescription, with specific details about the prescribing physician and the patient, in addition to elements of good practice, in addition to comprehensibility and avoiding abbreviations; the prescription also allows for detailed information,. Required fields included the name of the drug, dose, dosage form, route, frequency, and duration of treatment, in addition to sections regarding potentially high-risk groups (pregnant/breastfeeding women, elderly, and children), allergies, contraindications, and drug–drug interactions for poly-medicated patients.

#### Dispensing guidelines for pharmacists (details in Additional file 2) (25)

The main dispensing guidelines included sections on:

- the maximum validity of the prescription, set to one month after the date of issuing for acute conditions, and one year for chronic diseases.
- safe and appropriate treatment dispensing: the pharmacist should have an independent judgment and has to verify the prescription taking into account the following elements: drug dose, frequency, route of administration, duration of treatment, use of other medications, co-morbidities, medication history, and other relevant circumstances, including but not limited to allergies, compliance, and cost.
- medications counseling, including any oral or written information on dispensed medications to optimize the benefits of the therapy, improve patient’s understanding of the treatment, and enhance compliance while minimizing adverse effects.
- generic substitution.
- special and at-risk populations, e.g., pregnant/breastfeeding women, elderly, and children.
- dispensing safety check taking into account allergies, dosing, contraindications, drug-drug interactions, and potential side effects, before providing the medication to the patient.
- reporting errors and adverse drug reactions of any type.

#### Prescription format amendments (details in Additional file 3)

The OPL suggestions that would improve the functionality of the unified prescription include:

- using a triplicate prescription form: one copy for the patient, one for the pharmacist, and the third copy for the third-party payer (if available);
- adding essential missing fields as available through the electronic Lebanese Advanced Patient Profile, such as gender, age, and diagnosis and replacing the serial number of the prescription by a QR code (for better security and confidentiality);
- removing the medical laboratory tests section, and creating separate forms with no serial number for both medical laboratory tests and medical reports; those forms would be developed by concerned parties in collaboration with the MOPH.

#### Standard operation procedure for prescriptions workflow

In addition to these guidelines, standard operating procedures were suggested to improve the workflow of prescribing and dispensing medications (Additional file 4).

### Discussion

These guidelines are of primary importance, in the absence of any prior related recommendations in Lebanon. Guidelines for generating e-prescriptions are particularly important under the current COVID-19 pandemic circumstances and related confinement measures. Indeed, the COVID-19 outbreak has challenged all healthcare team members, including pharmacists who are required to secure adequate clinical pharmacy practice management, especially to high-risk patients such as those undergoing hematopoietic cell transplantation or cellular therapy [26]. Such a system would allow pharmacists to continue monitoring patients, providing them with specific education they might need during the pandemic. In that context, some already published recommendations about optimizing technology resources [26] can be added to the current guidelines. A recent published commentary described the “success story” of two Greek hospitals that allowed the continuity of care/therapies of patients with inflammatory bowel disease using telemedicine services, electronic-prescriptions, and home delivery of medications during the COVID-19 lockdown [27].

Moreover, although previous studies have shown that medication errors related to prescriptions were common in Lebanese hospitals [28, 29], and the community [30], the early stages of implementation of the medication safety system in Lebanon is in its infancy, as it is still in the early stages of implementation by the MOPH [31]. The suggested guidelines are thus expected to reduce the risk of prescription and dispensing errors, assess and decrease the risk of medications’ off-label use, decrease medication-related expenditures, promote better reporting of safety issues to authorities, and eventually improve patients’ health. The system might also help capture professional practice mal-adherence to clinical guidelines, potential lack of efficacy, which can, in turn, identify mis-prescribed medications, poor-quality medicines, or even clusters of antimicrobial resistance.

Hence, piloting these guidelines among a small sample of volunteer healthcare professionals would optimize those guidelines and confirm the value of the project before implementing it on a larger scale: The implementation of the guidelines should be formally evaluated, to assess if they achieve the stated short and long-term objectives to improve the quality of medicines' prescriptions and use, the patients care, and the communication among health care professionals, as well as to help rationalizing the pharmaceutical expenses.

## Conclusions

The Order of Pharmacists of Lebanon suggested guidelines and procedures for physicians and pharmacists in Lebanon to enhance the application of the Lebanese unified medical prescription; further assessment studies are necessary to confirm the value of this project in improving patients’ safety and optimizing the healthcare system in Lebanon.

## Supplementary information

**Additional file 1:** Prescription and E-prescription guidelines for physicians.

**Additional file 2:** Dispensing guidelines for pharmacists.

**Additional file 3:** Prescription format amendments.

**Additional file 4:** Suggested prescription workflow.

## Data Availability

All related data are attached as additional files.

## Abbreviations

MOPH: Lebanese Ministry of Public Health.
OPL: Order of Pharmacists of Lebanon.
WHO: World Health Organization
