# Supplementary material for: Prescription and dispensing guidelines in Lebanon: initiative of the Order of Pharmacists of Lebanon
Source: J Pharm Policy Pract. 2020 Nov 6;13:70. doi: 10.1186/s40545-020-00273-9 (PMC7644285; doi:10.1186/s40545-020-00273-9)
Supplement: Supplementary file 1 — Additional file 1: Prescription and E-prescription guidelines for physicians. [file 40545_2020_273_MOESM1_ESM.pdf]

December 18, 2017

PRESCRIPTION AND E-PRESCRIPTION GUIDELINES FOR PHYSICIANS

WORLD HEALTH ORGANIZATION PRESCRIPTION'S TIMELINE

STEP 1: Define the problem of the patient
STEP 2: Specify the therapeutic objective
STEP 3: Verify the suitability of a prescription drug
STEP 4: Write a prescription
STEP 5: Give information, instructions and warnings
STEP 6: Monitor and/or stop the treatment

BASIC LEGAL REQUIREMENTS OF A PRESCRIPTION

<u>Prescription should:</u>

- Be written clearly in ink to decrease risk of errors (for written prescriptions)
- Be indelible (including computer-generated prescriptions)
- Be signed and dated by the prescriber
- Be in e-prescription format to reduce prescribing errors and improve safety of dispensing
- Specify the third party payer when it exists

<u>Details pertaining to the prescribing physician should include:</u>

- Name, address and qualification of the prescriber
- Phone number
- Date of prescribing
- Stamp with physician's registration number

<u>Details pertaining to the patient should include:</u>

- Patient's full name
- Patient's age, weight and gender
- Patient's address and phone number

ELEMENTS OF GOOD PRACTICE

- Write generics (when available) unless there are bioavailability issues. This practice will enable the pharmacist to dispense any equally equivalent preparation, and avoid expenses and potential delays as per the decision flowchart on substitution on page 3.
- Strictly avoid abbreviations or scribbling the name of the medications.
- State name of drug, dose, dosage form, route, frequency and duration of treatment or any other useful information (empty stomach, with meals, after meals...)
- Specify the strength and quantity. In the absence of this information, the pharmacist will attempt to contact you. If unable to do so, the pharmacist can use their discretion and professional judgement to dispense up to five days' worth of treatment. If they have insufficient information to make a judgement, they will not dispense.
- Alterations are best avoided but if any is to be made, it should be clear, unambiguous and endorsed by the prescriber's signature.
- Avoid the unnecessary use of decimal points (trailing zero). For example, 3 grams should be written as 3g and not 3.0g, quantities of less than 1 gram should be written in milligrams (such as 500mg instead of 0.5g)

- When decimals are unavoidable, a zero should be written in front of the decimal point where there is no other figure e.g. 0.5ml, not .5ml. It is advisable to avoid trailing zeros (such as 0.50ml).
- Write refill information.
- Consider drug cost when prescribing.
- Draw a diagonal line across the blank part of the form under the prescription to prevent fraudulent alterations or additions being made.
- Schedules should preferably be written in English, without abbreviation, however some Latin abbreviations are acceptable (Table 1).

Table 1: Most Common Used Acceptable Latin Abbreviations		
Meaning	***French/American***	***English***
Immediately	*stat*	*stat*
When required	*prn* (pro re nata)*	*p.r.n.* (pro re nata)*
Every day	*qd (quaque die)*	*o.d. (omni die)*
Every night	*on*	*o.n. (omni nocte)*
Twice daily	*bid (bis in die)*	*b.d. (bis die)*
Three times daily	*tid (ter in die)*	*t.d.s. (ter die sumendum)*
Four times daily	*qid (quarter in die)*	*q.d.s. (quater die sumendum)*
Every so-many hours	*q_h (quaque_hora)*	
At bed time	*hs (hora somni)*	
**For prn dosage, state minimum dose interval and maximum total amount.*		

PRECAUTIONS WHEN WRITING A PRESCRIPTION

- Check allergy status of the patient.
- Check dosing, contraindications and potential side effects prior to issuing any prescription.
- Check what other medicines the patient is currently taking, including once weekly scheduled medications, inhalers, when needed medications and other prescription and non-prescription items, and supplements, which may interfere with the new medication.
- Exert caution in potential high-risk groups e.g. pregnancy/breastfeeding, elderly, children
- Advise patients of possible, likely and probable side effects and encourage them to report back any problems
- Respond promptly to a patient's concern/adverse drug reaction (ADR). Report it to the Lebanese Order of Pharmacists using the reporting form downloadable from (www.opl.org.lb/medicationsafety) and by returning it by mail to dic@opl.org.lb

GENERAL REMARKS

- In case the duration of treatment is not specified by the physician:
  - The maximum prescription validity for acute diseases is of 1 month starting the date of issuing
  - The maximum prescription validity for chronic diseases is of 1 year starting the date of issuing.
- In case refill information is lacking and the prescription is older than one month, no refill is allowed.

DECISION FLOWCHART ON SUBSTITUTION FOR PHARMACISTS

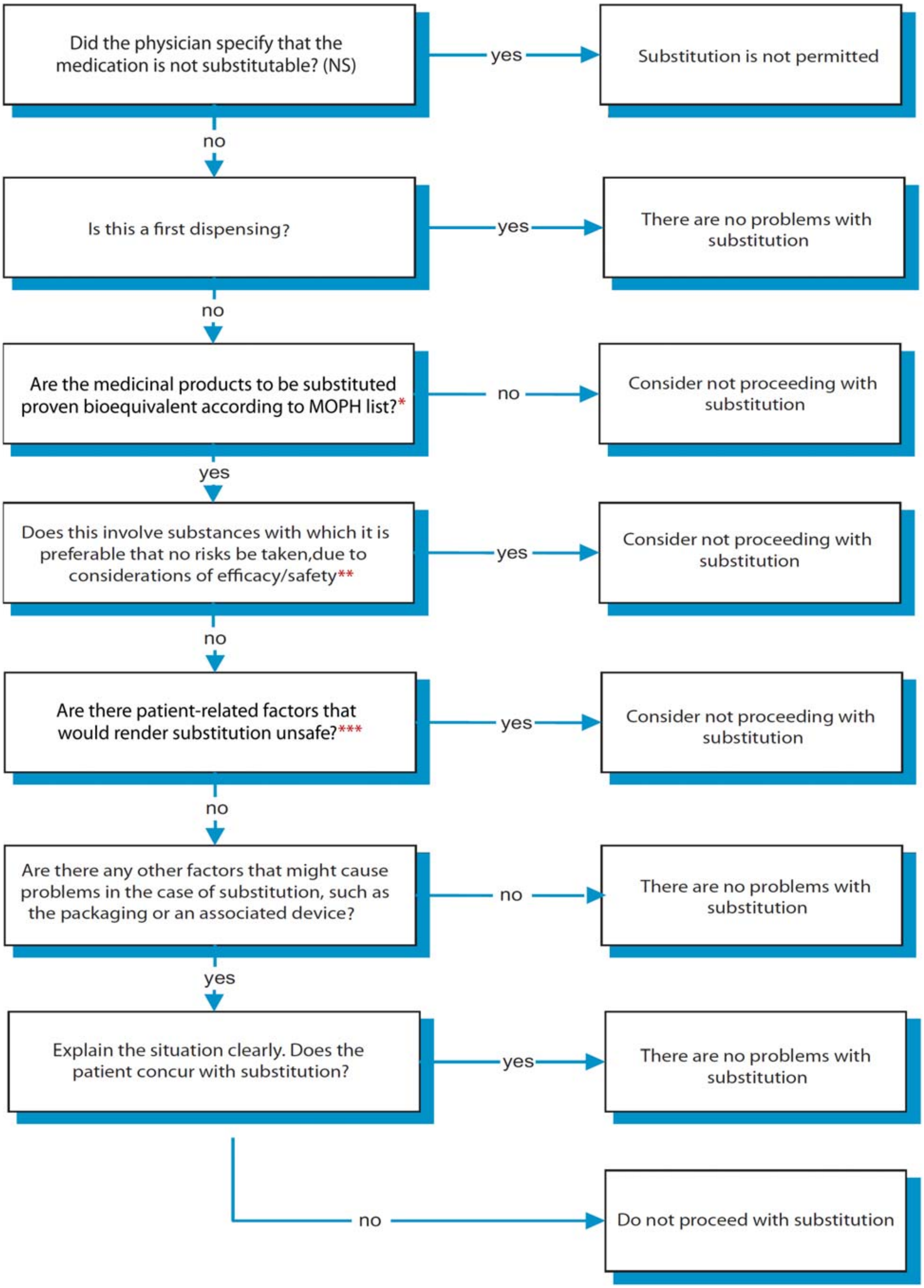

*: http://moph.gov.lb/userfiles/files/HealthCareSystem/Pharmaceuticals/LebanonSubstitutionDrugList/SubstitutionDrugListFE2015.pdf

**: medications with narrow therapeutic index, sustained release formulations and excipients with known effect

***: age, weight, organ dysfunction, altered mental status and medical critical condition

REFERENCES

Guide to Good Prescribing
http://apps.who.int/medicinedocs/pdf/whozip23e/whozip23e.pdf

How to Write a Prescription: Guidelines for Dentist
http://www.hse.ie/eng/services/list/2/gp/Antibiotic-Prescribing/Conditions-and-Treatments/Dental/Dental-prescription-guidelines/

General Prescribing Guidance Authored by Dr Mary Harding, 16 Jun 2014
https://patient.info/doctor/general-prescribing-guidance

Writing a Prescription: Guidelines
https://medicaldialogues.in/writing-a-prescription-guidelines/

Appropriate Prescribing of Medications: An Eight-Step Approach
http://www.aafp.org/afp/2007/0115/p231.html

Guideline for generic substitution – KNMP
https://www.knmp.nl/patientenzorg/geneesmiddelen/handleiding-geneesmiddelsubstitutie/at_download/file_4
