# Supplementary material for: Prescription and dispensing guidelines in Lebanon: initiative of the Order of Pharmacists of Lebanon
Source: J Pharm Policy Pract. 2020 Nov 6;13:70. doi: 10.1186/s40545-020-00273-9 (PMC7644285; doi:10.1186/s40545-020-00273-9)
Supplement: Supplementary file 2 — Additional file 2: Dispensing guidelines for pharmacists. [file 40545_2020_273_MOESM2_ESM.pdf]

December 18, 2017

DISPENSING GUIDELINES FOR PHARMACISTS

WORLD HEALTH ORGANIZATION PRESCRIPTION'S TIMELINE

**Physician**	STEP 1: Define the patient's problem<br>STEP 2: Specify the therapeutic objective<br>STEP 3: Verify the suitability of the prescription drug<br>STEP 4: Write the prescription
**Pharmacist**	STEP 5: Give information, instructions and warnings<br>STEP 6: Monitor the treatment (and stop, if necessary, in collaboration with the physician)

BASIC LEGAL REQUIREMENTS OF A PRESCRIPTION

Prescription should:

- Be written clearly in ink to decrease risk of errors (for written prescriptions)
- Be indelible (including computer-generated prescriptions)
- Be signed and dated by the prescriber
- Be in e-prescription format to reduce prescribing errors and improve safety of dispensing
- Specify the third party payer when it exists

Details pertaining to the prescribing physician should include:

- Name, address and qualification of the prescriber
- Phone number
- Date of prescribing
- Stamp with physician's registration number

Details pertaining to the patient should include:

- Patient's full name
- Patient's age, weight and gender
- Patient's address and phone number

MEDICAL INFORMATION REQUIRED IN A PRESCRIPTION

- The name of the medication, dose, dosage form, route, frequency and duration of treatment (including overall quantity) and other useful information (empty stomach, with meals, after meals...) should be stated. In the absence of this information, the pharmacist will attempt to contact the physician. If unable to do so, the pharmacist can use their discretion and professional judgement to dispense up to five days' worth of treatment. If they have insufficient information to make a judgement, they will refrain from dispensing the medication in question.
- Refill information
- Alterations are best avoided but if any is used, it should be clear, unambiguous and endorsed by the prescriber's signature.
- Schedules should preferably be written without abbreviation. However, some Latin abbreviations are acceptable (Table 1).

Table 1: Most Common Used Acceptable Latin Abbreviations		
Meaning	***French/American***	***English***
Immediately	*stat*	*stat*
When required	*prn* (pro re nata)*	*p.r.n.* (pro re nata)*
Every day	*qd (quaque die)*	*o.d. (omni die)*
Every night	*on*	*o.n. (omni nocte)*
Twice daily	*bid (bis in die)*	*b.d. (bis die)*
Three times daily	*tid (ter in die)*	*t.d.s. (ter die sumendum)*
Four times daily	*qid (quarter in die)*	*q.d.s. (quater die sumendum)*
Every so-many hours	*q_h (quaque_hora)*	
At bed time	*hs (hora somni)*	
**For prn dosage, state minimum dose interval and maximum total amount.*		

DISPENSING GUIDELINES FOR PHARMACISTS

- In case the duration of treatment is not specified by the physician:
  - The maximum prescription validity for acute diseases is of 1 month starting the date of issuing
  - The maximum prescription validity for chronic diseases is of 1 year starting the date of issuing.
- In case refill information is lacking and the prescription is older than one month, no refill is allowed.
- When dispensing a prescription, a pharmacist has to exercise an independent judgment to ensure that the medication is safe and appropriate for the patient, as well as it conforms to the prescriber's requirements. If there is any doubt, the prescriber is to be contacted.
- To verify the prescription, the pharmacist must take into account the following elements: drug dose, frequency and route of administration, duration of treatment, presence or absence of other medications, patient's illness, medication history, and other relevant circumstances including but not limited to allergies, compliance and cost.
- The simultaneous supply of multiple quantities of a particular medication (more than one refill at once) may not be in accordance with the prescriber's intention and is contrary to good pharmacy practice.
- An extemporaneous preparation should be used only in circumstances where a commercial product is unavailable or unsuitable.
- Errors during dispensing, omissions, incidents, or other (significant or not) and other non-compliances, including complaints of a non-commercial nature arising both within and external to the pharmacy, may be subject to investigation. Pharmacists should therefore keep appropriate records. Using the electronic patient profile platform suggested by the OPL is encouraged as it is a standardized solution for keeping records. It will also allow the OPL to conduct medication utilization reviews (MUR) for continuous improvement on patient care.
- Pharmacists should keep a scanned copy of any dispensed prescription when it will be received in hard copy format.
- Pharmacists are to give oral or written information on dispensed medications with the goal to: a) maximize the benefits of the therapy, b) improve the patient's understanding of the treatment, c) enhance compliance and minimizing adverse effects. [Label contents to be specified].

- Patients have the right to be counseled by the pharmacist about their medications (in a designated private area to maintain confidentiality), but the patient reserves the right not to be counseled. The pharmacist should make every effort to counsel the patient, or to offer counseling, whenever a medication is dispensed.
- Pharmacists should ensure that all pharmacy services are provided in a manner that respects the patient's privacy, and is in accordance with relevant professional and quality assurance standards.
- Adequate time must be allowed to dispense properly every prescription.
  - Routine checking throughout the dispensing process is necessary. It is important to emphasize the final check at the time of actual supply and when dispatching the medication to wards (when this is applicable).
  - Medications' counseling to patient or carer provides an additional check.
  - Distractions during the dispensing process have been linked to dispensing errors. Therefore, they should be minimized or made inexistent.
- Pharmacists should ensure that the pharmacy team (including pharmacy staff) is working in a good environment and under a manageable workload. This will help to:
  - Maximize patient safety.
  - Provide an appropriate pharmacy service in an accurate, professional and timely manner.
  - Cope with fluctuations in workflow.

ELEMENTS OF GOOD PRACTICE

- The pharmacist may substitute with generic medications (when available and when it is allowed as per the prescription) unless there are bioavailability issues, as per the decision flowchart on substitution for pharmacists on page 4). This practice will enable to: a) dispense any preparation deemed equivalent, and b) avoid expenses and potential delays.
- Consider drug cost when substituting.
- Check allergy status of the patient.
- Check dosing, contraindications and potential side effects prior to issuing any prescription.
- Check what other medicines the patient is currently taking, including once weekly scheduled medications, inhalers, when needed medications and other prescription and non-prescription items, and supplements, which may interfere with the new medication.
- Exert caution in potential high-risk groups e.g. pregnancy/breastfeeding, elderly, children
- Advise patients of possible, likely and probable side effects and encourage them to report any problems.
- Respond promptly to a patient's concern/adverse drug reaction (ADR). Report it to the Lebanese Order of Pharmacists: www.opl.org.lb/medicationsafety

SPECIAL CONSIDERATIONS

- A pharmacist is allowed to dispense a prescription medication (including psychotropics but not narcotics), based on an oral prescription in life-saving situations or upon the discretion and judgement of the physician. Pharmacist will:
  - Make a copy of the patient's ID and make him sign an informed consent
  - Call the physician and take the approval to dispense.
- Physician will give the pharmacist the serial number of the prescription along with his OML ID and send it electronically or physically to the pharmacist.
- Patient will have a week to bring the prescription.
- Non-Prescription Medications can be dispensed as per an updated list, approved by the Ministry of Public Health.

DECISION FLOWCHART ON SUBSTITUTION FOR PHARMACISTS

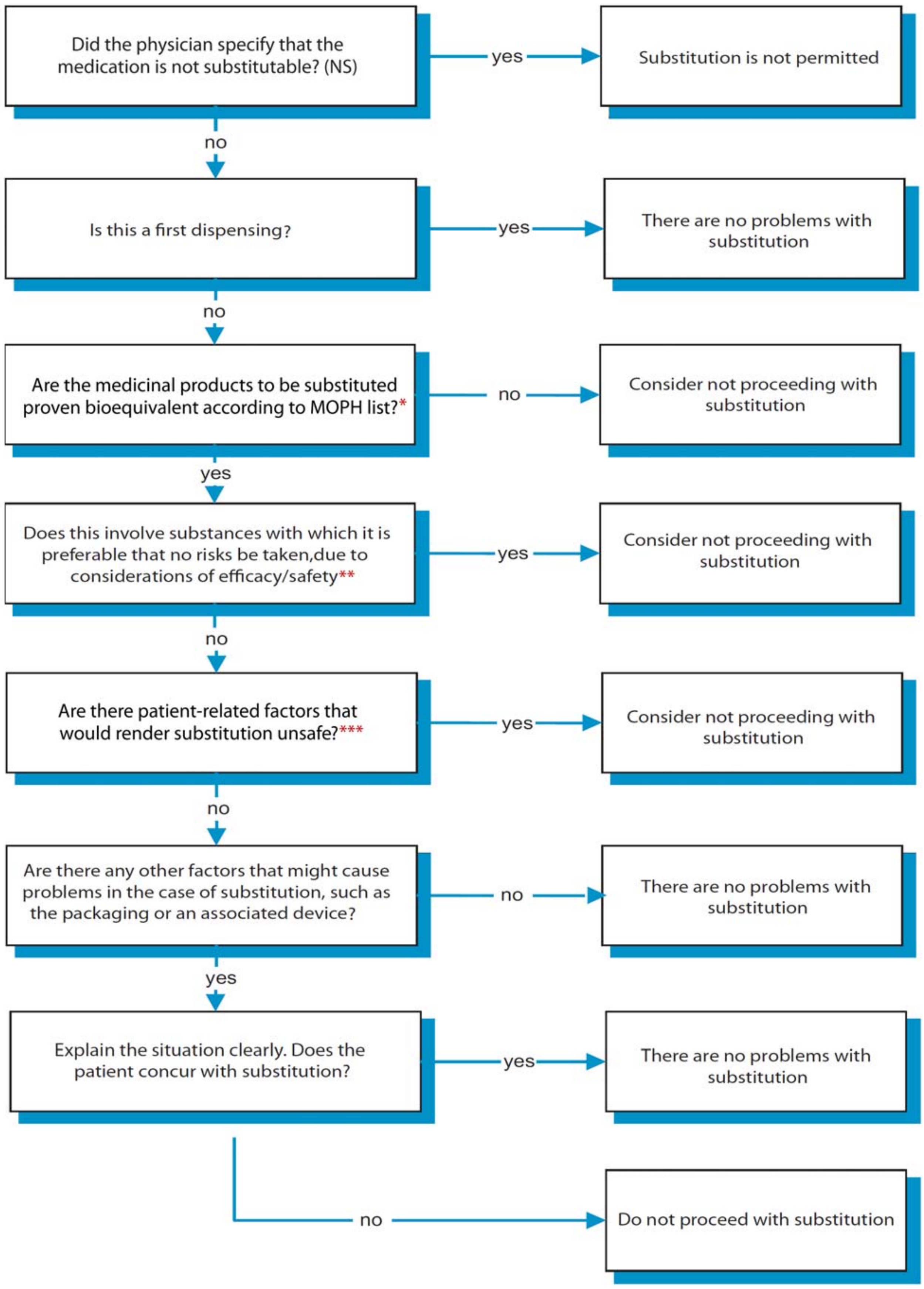

*: http://moph.gov.lb/userfiles/files/HealthCareSystem/Pharmaceuticals/LebanonSubstitutionDrugList/SubstitutionDrugListFE2015.pdf

**: medications with narrow therapeutic index, sustained release formulations and excipients with known effect

***: age, weight, organ dysfunction, altered mental status and medical critical condition

REFERENCES

Guide to Good Prescribing
http://apps.who.int/medicinedocs/pdf/whozip23e/whozip23e.pdf

Guidelines for Dispensing of Medicines
- http://www.pharmacyboard.gov.au/documents/default.aspx?record=WD10%2F2951&dbid=AP&chksum=WMyYdhKfX3%2BWGPiGUCLsMw%3D%3D
- http://apps.who.int/medicinedocs/en/d/Js17807en/

How to Write a Prescription: Guidelines for Dentist
http://www.hse.ie/eng/services/list/2/gp/Antibiotic-Prescribing/Conditions-and-Treatments/Dental/Dental-prescription-guidelines/

General Prescribing Guidance Authored by Dr Mary Harding, 16 Jun 2014
https://patient.info/doctor/general-prescribing-guidance

Writing a Prescription: Guidelines
https://medicaldialogues.in/writing-a-prescription-guidelines/

Appropriate Prescribing of Medications: An Eight-Step Approach
http://www.aafp.org/afp/2007/0115/p231.html

Guideline for generic substitution – KNMP
https://www.knmp.nl/patientenzorg/geneesmiddelen/handleiding-geneesmiddelsubstitutie/at_download/file_4
