# Supplementary material for: Prescription and dispensing guidelines in Lebanon: initiative of the Order of Pharmacists of Lebanon
Source: J Pharm Policy Pract. 2020 Nov 6;13:70. doi: 10.1186/s40545-020-00273-9 (PMC7644285; doi:10.1186/s40545-020-00273-9)
Supplement: Supplementary file 3 — Additional file 3: Prescription format amendments. [file 40545_2020_273_MOESM3_ESM.pdf]

نقابة صيادلة لبنان
ORDRE DES PHARMACIENS DU LIBAN

Certified System
Quality
ISO 9001
SAI GLOBAL

January 11, 2018

PRESCRIPTION FORMAT AMENDMENTS

The below suggestions would improve the functionality of the unified prescription:

1. Triplicate prescription form:
   - First paper: white color paper (no carbon-copy), written and stamped by the physician and filed by the pharmacist.
   - Second paper: duplicate white color carbon copy paper holding the mention "DUPLICATE. NOT SUITABLE FOR DISPENSING", written and stamped by the physician and used by the patient to get reimbursed by third party payers.
   - Third paper: triplicate and carbon copy of the duplicate (2nd paper), any color, to be filed by the physician.

2. Add important missing fields as available through the Lebanese Advanced Patient Profile:
   - Change the serial number of the prescription to a QR code (for a better security and confidentiality)
   - Gender
   - Age
   - Diagnosis

3. The laboratory part should be on a separate unnumbered* form, to be developed by the "Syndicat des Biologistes au Liban" with the collaboration of the MOPH.

4. The medical report should be written on a separate unnumbered* form, to be developed by the "Lebanese Order of Physicians" with the collaboration of the MOPH.

** This would prevent the physician from paying taxes twice or more for the same patient.*

• Corniche du Fleuve, immeuble Caisse de Retraite des Pharmaciens • B.P: 11-2807 Beyrouth - Liban •
• Téléphone: +961 1 611344 • Télécopie: +961 1 611342 • Courriel: dic@opl.org.lb •
