# Supplementary material for: Prescription and dispensing guidelines in Lebanon: initiative of the Order of Pharmacists of Lebanon
Source: J Pharm Policy Pract. 2020 Nov 6;13:70. doi: 10.1186/s40545-020-00273-9 (PMC7644285; doi:10.1186/s40545-020-00273-9)
Supplement: Supplementary file 4 — Additional file 4: Suggested prescription workflow. [file 40545_2020_273_MOESM4_ESM.pdf]

February 16, 2018

SUGGESTED PRESCRIPTION WORKFLOW

1. PHYSICIAN ISSUES A HARD-COPY PRESCRIPTION:

- Physician issues the prescription in 3 copies all stamped and signed:
  - First paper: white color paper (no carbon-copy), written and stamped by the physician and filed by the pharmacist.
  - Second paper: white color carbon copy paper holding the mention "DUPLICATE. NOT SUITABLE FOR DISPENSING", written and stamped by the physician and used by the patient to get reimbursed by third party payers.
  - Third paper: carbon copy of the 2nd paper, any color, to be filed by the physician.
- Patient presents with a 2-copy prescription
  - Pharmacist enters content of the prescription on the platform, scans it and files the white and stamped copy (original).
  - Pharmacist prints out a receipt for refill purposes and a label (or a paper) with the physician's directions on how to use the medications
- Pharmacist dispenses medications, counsels on the appropriate use of prescribed medications and checks on interactions
- Pharmacist can do further counseling and follow-up by filling the patient profile after getting patient's approval

2. PHYSICIAN FILLS THE PRESCRIPTION ELECTRONICALLY:

- Physician fills the prescription electronically and gets an automatic serial number or QR code. The prescription is stored on the server.
- Two options are available to be presented to the pharmacist:
  - The patient receives a printed label with the serial number of the prescription and the phone number he gave to the physician to fill out the prescription
  - The patient receives an SMS on his mobile phone with the serial number of the prescription or a link to the QR code of the prescription
- Pharmacist prints out for the patient:
  - Labels (with physician's directions on how to use the medications)
  - A receipt for refill purposes
  - NSSF receipt if applicable

- Pharmacist submits electronic stamped copies to:
  - The MOPH (automatically for all transactions)
  - The NSSF and/or Third Party Payer (as directed by the pharmacist)
- Pharmacist dispenses medications, counsels on the appropriate use of prescribed medications and checks on interactions
- Pharmacist can do further counseling and follow-up by filling the patient profile after getting patient's approval
- Pharmacist should collaborate with the physician when needed
